# Long-Term Sleep Duration as a Risk Factor for Breast Cancer: Evidence from a Systematic Review and Dose-Response Meta-Analysis

**DOI:** 10.1155/2017/4845059

**Published:** 2017-10-10

**Authors:** Chunyang Lu, Hao Sun, Jinyu Huang, Songcheng Yin, Wenbin Hou, Junyan Zhang, Yanshi Wang, Yingying Xu, Huimian Xu

**Affiliations:** ^1^Department of Surgical Oncology and General Surgery, The First Hospital of China Medical University, No. 155 Nanjing North Street, Heping District, Shenyang, Liaoning Province 110001, China; ^2^Department of Clinical Epidemiology and Evidence-Based Medicine, The First Hospital of China Medical University, No. 155 Nanjing North Street, Heping District, Shenyang, Liaoning Province 110001, China; ^3^Department of Gynaecology, The First Hospital of China Medical University, No. 155 Nanjing North Street, Heping District, Shenyang, Liaoning Province 110001, China; ^4^Department of Breast Oncology and General Surgery, The First Hospital of China Medical University, No. 155 Nanjing North Street, Heping District, Shenyang, Liaoning Province 110001, China

## Abstract

Sleep patterns have been associated with the development of cancers, although the association between sleep duration and breast cancer remains controversial. The purpose of our study was to explore the relationship between sleep duration and breast cancer risk. The PubMed and Web of Science databases were searched, and restricted cubic splines were used to explore the dose-response relationship. Data from 415,865 participants were derived from 10 studies. A J-shaped nonlinear trend was found between sleep duration and breast cancer incidence (*P*_non-linear_ = 0.012); compared with the reference hours (6 h or 7 h), with increasing sleep hours, the risk of breast cancer increased (*P*_trend_ = 0.028). Moreover, a nonlinear relationship was found between sleep duration and estrogen receptor-positive breast cancer (*P*_non-linear_ = 0.013); the risk of estrogen receptor-positive breast cancer increased with increasing sleep hours compared to the reference hours (*P*_trend_ = 0.024). However, no nonlinear relationship was found between sleep duration and estrogen receptor-negative breast cancer; the risk of estrogen receptor-negative breast cancer was 1.035 for every additional sleep hour. Compared to women with the reference number of sleep hours, women with a longer sleep duration might have a significantly increased risk of breast cancer, especially estrogen receptor-positive breast cancer.

## 1. Introduction

Globally, breast cancer is the most frequently diagnosed cancer and the leading cause of cancer mortality among women. The incidence of breast cancer increases by 5 percent each year in low- and middle-income countries and thus represents an increasingly urgent public health problem [1, 2]. Many studies have focused on risk factors for breast cancer. For instance, the commentary by Colditz established reproductive characteristics, growth, obesity, and postmenopausal hormones as significant etiological factors, although these variables accounted for just a small proportion of breast cancer risk [3]. With societal development, attention has increasingly been turned to quality of life and lifestyle factors, such as sleep patterns, night-shift work, alcohol consumption, and weight gain, as important influencing factors that may provide additional clues for efforts to prevent breast cancer.

Recently, sleep patterns (including sleep duration or sleep quality) and their relationships with related health outcomes have been studied, mostly regarding chronic diseases such as cardiovascular diseases, diabetes, obesity, and metabolic syndrome [4–7]. As a possible mechanism, sleep might affect the levels of circulating hormones, such as melatonin, cortisol, growth hormone, prolactin, glucose, and insulin, which are key factors involved in many disease processes, including breast cancer [8]. Thus, a growing number of epidemiologic studies have examined the association between sleep duration and breast cancer incidence; however, the results have been inconsistent [9–12]. Therefore, we performed this updated dose-response meta-analysis to explore the association between sleep duration and breast cancer incidence, which might provide some insight and evidence for efforts to prevent breast cancer in women.

## 2. Materials and Methods 

This meta-analysis was conducted according to the meta-analysis of observational studies in epidemiology (MOOSE) checklist [19].

### 2.1. Literature Search Strategy

Two authors (Chunyang Lu and Hao Sun) conducted a computerized search for published articles in the PubMed and Web of Science databases from inception to September 2nd, 2016, without language restrictions. Discrepancies were resolved through discussion with other authors (Jinyu Huang and Songcheng Yin). The following search terms were used: (1) breast cancer OR mammary cancer OR breast neoplasms OR mammary neoplasms; (2) sleep OR sleep duration OR sleep pattern. We also screened the reference lists of relevant review articles and included studies for additional information.

### 2.2. Selection Criteria

Studies were included only if they met all the following criteria: (1) the study was an observational study (case-control study or cohort study); (2) the outcome was the first incidence of breast cancer; (3) the exposure was sleep duration with at least three categories; and (4) there were available effect estimates (risk ratios (RRs)) or odds ratios (ORs) and 95% confidence intervals and the corresponding number of cases and person-years or subjects for each category of sleep duration. Comments, reviews, letters, animal experiments, and studies involving other cancers were excluded. If multiple publications were available for a study, data from the most recent and complete publication were included.

### 2.3. Data Extraction

Two authors (Chunyang Lu and Hao Sun) evaluated study eligibility and conducted data extraction independently using a predefined standardized data extraction form. The variables in the form included the following: name of the first author, publication year, study country, study name, study design, follow-up year (if cohort study), investigation year (if case-control study), age at baseline, the proportion of postmenopausal participants, method of sleep data collection, the ascertainment of breast cancer, sample size (numbers of participants and incident cases) in each category, covariates adjusted in the multivariable analysis, and effect size (RRs or ORs) with a 95% CI for all categories of sleep duration.

### 2.4. Quality Assessment

Quality assessment was performed according to the Newcastle-Ottawa Quality Assessment Scale (NOS) [20], which is a validated scale for nonrandomized studies in three areas: the selection of exposed and unexposed participants; the comparability of the groups; and the assessment of the outcome. This tool contains nine items, with each item being assigned a star if a study meets the criteria for the item. We considered a study as high-quality if it received 7 or more stars.

### 2.5. Statistical Analysis

First, we conducted restricted cubic splines with five knots at the 1st, 25th, 50th, 75th, and 99th percentiles of exposure distribution to fit the potential nonlinear trend between sleep duration and incidence of breast cancer. In this method, the distributions of cases and participants and the fully adjusted RRs or ORs and 95% CIs in each sleep duration category were required. For studies that did not report the numbers of breast cancer cases for each category of sleep duration, these numbers were inferred based on the total numbers of cases and the reported risk estimates. Additionally, for each of the included studies, we assigned the reported median or mean sleep duration of each category as the category sleep duration. When a study reported only the range of sleep duration for a category, we used the average value of the lower and upper bounds of that category. When the shortest or the longest category was open-ended, we assumed that the open-ended interval length had the same length as the adjacent interval. The reference category was set to 6 h (the lowest risk of breast cancer in our study based on the dose-response curve) or 7 h (international sleep duration recommendation) [21]. A *P* value for nonlinearity was calculated by testing the null hypothesis that the coefficient of the second and third spline was equal to 0. If the hypothesis did not hold, we conducted a linear dose-response meta-analysis to test the risk of breast cancer with each additional hour of sleep. Otherwise, the nonlinear dose-response meta-analysis was conducted to show each hour's risk of breast cancer (from 4 h to 10 h). We also performed subgroup analyses stratified by menopausal status (postmenopausal or premenopausal) and estrogen receptor (ER) status (ER+ or ER−). All analyses were performed with STATA version 12.0 (StataCorp, College Station, TX), and all tests were two-sided with a significance level of 0.05.

## 3. Results

### 3.1. Literature Search and Characteristics of the Included Studies

**Figure 1:**
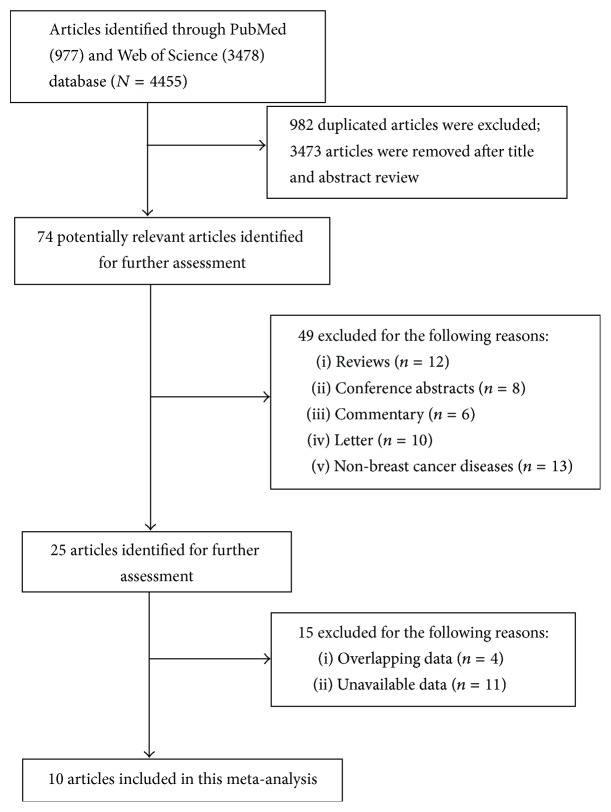
Flow diagram for studies selection.

A total of 10 studies [9–18] composed of six cohort studies [9, 10, 12, 15, 16, 18], three case-control studies [11, 14, 17], and one nested case-control study [13] were included in our final meta-analysis. The details of how we selected relevant studies are shown in Figure 1.

The characteristics of the studies and participants included in this study were presented in Tables 1 and 2. Of these studies, five were conducted in America [9, 10, 12, 16, 17], three were conducted in Asia [11, 13, 15], one was conducted in Australia [14], and one was conducted in Europe [18]. The sample sizes ranged from 1,454 to 110,011, and the proportion of postmenopausal women in most of the studies was over 50%, except one study with 37.14% [11] and one study that did not report menopausal status [18]. The sleep durations were all self-reported via questionnaire or interview; the sleep reference categories in each study were set to ≤6 h [13], 7 h [12, 15, 16], 8 h [9, 18], 6.1 to 8.9 h [11], 7 to 7.9 h [17], 7 to 8 h [14], and 7 to 9 h [10]. The ascertainment of breast cancer was mostly through cancer registry or medical records. Most studies were adjusted for a wide range of potential confounders, such as menopausal status, body mass index (BMI), smoking, and alcohol drinking. The qualities of the individual studies were listed in Table 2.

### 3.2. Association between Sleep Duration and Breast Cancer

**Figure 2:**
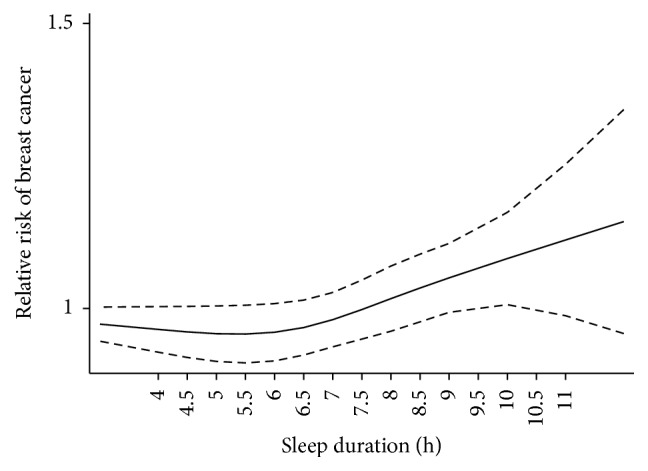
The plot of dose-response relationship between sleep duration and breast cancer risk.

**Figure 3:**
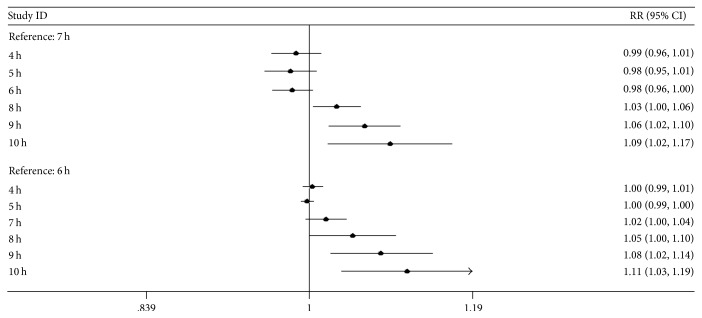
The trend of relationship between sleep duration and breast cancer risk.

A J-shaped nonlinear trend between sleep duration and breast cancer incidence was shown in Figure 2 (*P*_non-linear_ = 0.012). For the overall analysis, we used a fixed-effects model because there was no significant within-study heterogeneity (*P*_heterogeneity_ = 0.064). Because there was a nonlinear trend, we conducted a nonlinear dose-response meta-analysis to evaluate each additional sleep hour's risk of breast cancer. As Table 3 and Figure 3 show, compared with the reference hours (6 h or 7 h), with increasing sleep hours, the risk of breast cancer increased, and the increasing trend was significant (*P*_trend_ = 0.028).

### 3.3. Association between Sleep Duration and Breast Cancer according to Menopausal Status

Four studies [11, 14–16] and five studies [11, 13–16] reported the dose-response data in premenopausal and postmenopausal populations, respectively. For the nonlinear tests, we did not find nonlinear relationships between sleep duration and breast cancer risk in either premenopausal women or postmenopausal women (*P*_non-linear_ = 0.139; *P*_non-linear_ = 0.298). Therefore, we conducted a linear dose-response meta-analysis. The results showed that the relative risk (RR) of breast cancer was 1.012 in the premenopausal population and 1.003 in the postmenopausal population for every additional sleep hour; however, neither showed statistical significance (*P*_trend_ = 0.198; *P*_trend_ = 0.506).

### 3.4. Association between Sleep Duration and Breast Cancer according to Estrogen Receptor (ER) Status

**Figure 4:**
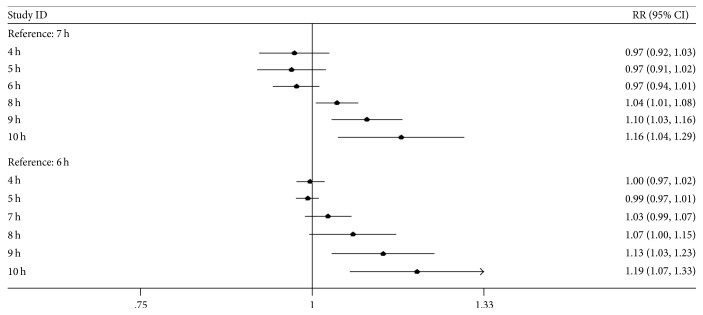
The trend of relationship between sleep duration and ER+ breast cancer risk.

Three studies [9, 11, 12] reported dose-response data on ER+ and ER− breast cancer. According to the nonlinear test, there was a nonlinear relationship between sleep duration and ER+ breast cancer (*P*_non-linear_ = 0.013) and a linear relationship between sleep duration and ER− breast cancer (*P*_non-linear_ = 0.139). As shown in Table 3 and Figure 4, the risk of ER+ breast cancer increased with increasing sleep hours compared with the reference hours, and the increasing trend was significant (*P*_trend_ = 0.024). Regarding ER− breast cancer, a linear dose-response meta-analysis showed that the RR of ER− breast cancer was 1.035 for every additional sleep hour (*P*_trend_ = 0.352).

## 4. Discussion

Our meta-analysis included 10 studies involving 415,865 participants to reliably quantify the association between sleep duration and breast cancer risk. An increased risk trend was found between sleep duration and breast cancer; our study also indicated that, compared to women with a normal sleep duration, women with a longer sleep duration might have a significantly increased risk of breast cancer; this was not observed among women with a shorter sleep duration. Subgroup analysis for breast cancer by ER status also showed a positive association between sleep duration and the risk of ER+ breast cancer, although this was not observed for ER− breast cancer.

Before 2008, most published articles involving sleep duration and breast cancer risk reported null findings [15–18] or that shorter sleep duration might be a risk factor [15] and longer sleep duration might be a preventative factor for breast cancer [15, 16, 18], which is inconsistent with our findings. However, after 2008, an increasing number of studies have reported slightly positive associations between longer sleep hours and breast cancer, in agreement with the findings in our meta-analysis. In the US case-control study, a positive trend for breast cancer was found for every additional sleep hour (OR = 1.06, 95% CI = 1.01~1.11) [13], and another study observed a positive association between sleep duration and breast cancer incidence with a RR of 1.58 (95% CI = 1.18~2.12) for 9 h or more compared to the reference hours (6.1 h to 8.9 h) [11]. Among the studies of breast cancer by ER status, Vogtmann et al.'s study observed a positive trend for increasing sleep duration with the risk of ER+ breast cancer [12], and Wang et al.'s study reported that longer sleep might be a risk factor for ER+ breast cancer compared to reference hours [11], which is consistent with our findings. Due to controversial results, four meta-analyses of the relationship between sleep duration and breast cancer have been previously published [22–25]; most of these articles pooled the results using a traditional two-category model. The most recent meta-analysis was published in 2012 with only six original articles included (four articles published before 2008) and found no significant association between either short or long sleep duration and breast cancer risk [22]. Therefore, our study might provide the most comprehensive assessment and robust evidence to evaluate the relationship between sleep duration and breast cancer risk.

The positive association between sleep duration and risk of breast cancer and ER+ breast cancer in our study was unexpected, as the melatonin hypothesis suggests that a short sleep duration is associated with decreased levels of melatonin, and melatonin is known to regulate susceptibility to cancer and to have antiproliferative activity [26]. However, Wu et al.'s research suggested that sleep duration might be inversely associated with urinary melatonin levels (aMT6s levels were 1.88, 1.85, 1.23, and 1.32 for ≤6, 7, 8, and ≥9 h of sleep, resp.; *P*_trend_ = 0.018) [13]. In addition, lower melatonin levels were correlated with higher estrogen levels [27], and increased estrogen exposure has been strongly linked to ER+ breast cancer [28, 29], which indicates that a link between longer sleep and breast cancer is biologically plausible. Additionally, inflammation was also considered to initiate and promote breast cancer development. Indeed, some authors believed that excessive sleep might lead to elevated levels of systemic inflammation and increase some inflammatory biomarkers, such as CRP and IL-6, which might predispose an individual to breast cancer [30, 31]. Furthermore, longer sleep might also increase the breast cancer risk through increased levels of cortisol and reductions in natural killer cell activity [32, 33].

There were several limitations of our study. First, misclassification bias might exist in our study because sleep duration was self-reported in the included studies. Compared with wrist actigraphy, which was an objective measure of sleep, self-reported sleep duration showed poor validation, and the sleep duration reported in questionnaires tended to be longer than the measured duration [34, 35]. However, subjectively measured sleep is the only practical option in large population studies. Second, the assessment of sleep duration in most studies was based on different time scales, which might influence the accuracy of our results. Third, other aspects of sleep such as snoring, sleep quality, and sleep disorder diseases that might influence breast cancer were not considered in our included studies. Indeed, studies have reported that long-term sleep duration might reflect poor sleep quality [36], and poor sleep quality might be related to cancer development. Even with the above-mentioned limitations, our study had several strengths. The mean Newcastle-Ottawa Scale (NOS) score of our study was 7.6, which means that the quality of the included studies was high and the results were reliable. Moreover, most included studies had adequately adjusted for potential confounders to reduce their possible impact on the association between sleep duration and breast cancer. Third, we used restricted cubic splines to fit potential dose-response trends, which made full use of the available data and had higher statistical power than traditional binary meta-analysis. Therefore, our meta-analysis might provide useful and robust evidence to evaluate the relationship between sleep duration and breast cancer.

## 5. Conclusions

In conclusion, our dose-response meta-analysis indicates that women with longer sleep durations might have a significantly increased risk of breast cancer, especially of ER+ breast cancer, compared to that of women with a normal sleep duration.

## Figures and Tables

**Table 1:** Characteristics of studies and participants included in meta-analysis.

Author	Year	Country	Study	Number of participants	Follow-up/investigated years	Age at baseline	Postmenopausal (%)	Study design
Xiao et al. [9]	2016	America	Southern community cohort study	42953	2002–2009	52.18	66.70%	Cohort study

Hurley et al. [10]	2015	America	California teachers study	101609	“1995-1996” to 2011	52.00	58.28%	Cohort study

Wang et al. [11]	2014	China	Guangzhou breast cancer study	1454	2010–2012	47.50	37.14%	Case-control study

Vogtmann et al. [12]	2013	America	Women's health initiative	110011	1992–2007	62.91	100%	Cohort study

Wu et al. [13]	2012	Singapore	Singapore Chinese health study	34028	“1993–1998” to 2010	45–74	92.73%	Nested case-control study

Girschik et al. [14]	2012	Australia	Breast cancer environment and employment study	2828	2009–2011	18–80	88.79%	Case-control study

Kakizaki et al. [15]	2008	Japan	Ohsaki national health insurance cohort study	23995	1994 to “1995–2003”	60.76	65.42%	Cohort study

Pinheiro et al. [16]	2006	America	Nurses' health study	77418	“1986, 2000” to “1986, 2002”	52.94	56.87%	Cohort study

McElroy et al. [17]	2005	America	US case-control study	9347	1997–1999	20–69	54.98%	Case-control study

Verkasalo et al. [18]	2005	Finland	Finnish twin cohort	12222	“1975, 1981” to “1976–1996”	36.50	—	Cohort study

**Table 2:** The outcome and related information included in this meta-analysis.

Author	Year	Data collection	Breast cancer ascertainment	Sleep duration (h/d) and risk (95% CI)	Adjusted variable	Quality assessment
Xiao et al. [9]	2016	Questionnaire	State cancer registries; pathology reports; medical records	<6: 1.09 (0.83–1.44) 6: 1.01 (0.78–1.30) 7: 1.19 (0.92–1.53) 8: reference ≥9: 1.07 (0.80–1.43)	Age, enrollment year, enrollment state, race, education, income, marital status, BMI, physical activity, overall sitting, smoking, pack-year, number of live birth, age at first birth, length of breastfeeding, age at menarche, menopause status, hormone therapy, multivitamin, aspirin, diabetes, family history of cancer, alcohol consumption, and dietary intakes of total fat, fiber, folate and total calories	8

Hurley et al. [10]	2015	Questionnaire	California cancer registry	3–6: 0.97 (0.91–1.04) 7–9: reference ≥10: 1.13 (0.86–1.50)	Race/ethnicity, alcohol consumption, menopausal status, hormone therapy use	6

Wang et al. [11]	2014	Face-to-face interview	Medical records	≤6: 1.62 (1.18–2.21) 6.1–8.9: reference ≥9: 1.58 (1.18–2.12)	Age, education, BMI, age at menarche, menopausal status, parity, physical activity, breastfeeding, family history of breast cancer, other sleep factors	7

Vogtmann et al. [12]	2013	Interview	Pathology reports; medical records	≤5: 0.89 (0.80–1.00) 6: 0.92 (0.85–0.98) 7: reference 8: 1.00 (0.93–1.07) ≥9: 1.05 (0.92–1.20)	Age, clinical trial arm assignment, number of live births, age at menarche, age at menopause, BMI, energy expenditure, education, income, race/ethnicity, marital status, age at first birth, use of hormone therapy, history of benign breast disease, family history of breast cancer, alcohol consumption, smoking status	8

Wu et al. [13]	2012	In-person interview	National cancer registry	≤6: reference 7: 1.00 (0.84–1.19) 8: 1.00 (0.84–1.21) ≥9: 0.89 (0.64–1.22)	Age, year, dialect group, education, parity, BMI, menopausal status	8

Girschik et al. [14]	2012	Self-administered postal questionnaire	Western Australian cancer registry	<6: 1.04 (0.83–1.32) 6-7: 0.95 (0.79–1.14) 7-8: reference >8: 1.05 (0.83–1.33)	Age, number of children, age at first birth, breastfeeding, menopausal status, use and duration of hormone therapy, alcohol consumption, comparative weight at the age of 30 years, melatonin, physical activity	8

Kakizaki et al. [15]	2008	Questionnaire	Miyagi prefectural cancer registry	≤6: 1.67 (1.002–2.78) 7: reference 8: 0.99 (0.59–1.65) ≥9: 0.29 (0.09–0.98)	Age, BMI, history of disease, family history of cancer, marital status, education, alcohol consumption, time spent walking, caloric intake, menopausal status, age at menarche, age at first delivery, number of deliveries, oral contraceptive drugs, hormone drug	8

Pinheiro et al. [16]	2006	Mailed questionnaire	Blinded medical chart review	≤5: 0.93 (0.79–1.09) 6: 0.98 (0.91–1.06) 7: reference 8: 1.05 (0.97–1.13) ≥9: 0.95 (0.82–1.11)	Age, BMI, height, history of benign breast disease, family history of breast cancer, parity and age at first birth, age at menarche, postmenopausal hormone use, physical activity, alcohol, caloric intake, smoking	7

McElroy et al. [17]	2005	Telephone interviews	State cancer registries	<5: 0.94 (0.62–1.44) 5–5.9: 0.82 (0.65–1.05) 6–6.9: 0.89 (0.79–1.01) 7–7.9: reference 8–8.9: 1.01 (0.91–1.11) ≥9: 1.01 (0.84–1.23)	Age, state, parity, age at first full-term pregnancy, family history of breast cancer, alcohol consumption, BMI, menopause status, age at menopause, postmenopausal hormone use, marital status	8

Verkasalo et al. [18]	2005	Questionnaires	Finnish cancer registries	≤4: 0.88 (0.11–6.91) 5: 0.91 (0.36–2.32) 6: 0.74 (0.44–1.27) 7: 0.81 (0.60–1.10) 8: reference 9: 0.64 (0.40–1.02) ≥10: 0.65 (0.24–1.75)	Age, zygosity, social class, number of children, use of oral contraceptives, BMI, alcohol use, smoking, physical activity	8

**Table 3:** The relative risks (RRs) for nonlinear and linear model.

	Number of studies	*P*_heterogeneity_	*P*_non-linear_	RR									*P*_trend_
				Nonlinear								Linear	
				4 h	5 h	6 h	7 h	8 h	9 h	10 h		1 h increment	
All	10	0.064	0.012	0.986	0.980	0.982	1.000	1.03	1.061	1.091		—	**0.028**
				1.004	0.998	1.000	1.018	1.049	1.08	1.111			
													
Premenopausal	4	0.132	0.139	—								1.012	0.198
Postmenopausal	5	0.241	0.298	—								1.003	0.506
													
ER+	3	0.705	0.013	0.971	0.966	0.974	1.000	1.042	1.096	1.16		—	**0.024**
				0.997	0.992	1.000	1.027	1.070	1.126	1.192			
ER−	3	0.018	0.139	—								1.035	0.352
